# The Hydrostatic Test of Stillbirth

**Published:** 1900-12-15

**Authors:** 


### THE HYDROSTATIC TEST OF STILLBIRTH.

Dr. Dilworth [1] records a case which shows how careful one must be in accepting the trustworthiness of the "hydrostatic test" of stillbirth, at any rate, when some days have elapsed before the examination of the body has been made. Owing to some rumours an inquest was held on the body of an illegitimate female child, which was exhumed after being buried ten days. The evidence given at the inquest went to show that the child had been born rather precipitately, but that the woman had been attended first by some neighbouring women, then by a medical man, and finally by the parish nurse, all of whom saw the baby alive, though weakly. At the *post-mortem* the body was found to present the appearance of a child born at full term and fairly well developed. On opening the chest the lungs were found collapsed, not filling the chest cavity, and in a state of complete atelectasis. On resort being had to the hydrostatic test it was found that the lungs sank when both were immersed together, when immersed separately, and even when small portions were thrown in —in fact, they presented all the appearances found in the lungs of a stillborn child. The matter seems of some interest as emphasising the doubt which has often been expressed as to the reliability of this sign of stillbirth.

[1] Brit. Med. Jour., Dec. 1.

